# Viremic long-term nonprogressive HIV-1 infection is not associated with abnormalities in known Nef functions

**DOI:** 10.1186/1742-4690-11-13

**Published:** 2014-02-04

**Authors:** Anke Heigele, David Camerini, Angélique B van’t Wout, Frank Kirchhoff

**Affiliations:** 1 Institute of Molecular Virology, Ulm University Medical Center, Ulm, Germany; 2 Cancer Research Institute, Institute for Immunology, Center for Virus Research, University of California Irvine, Irvine, California, USA; 3 Department of Experimental Immunology, Sanquin Research, Landsteiner Laboratory and Center for Infection and Immunity Amsterdam (CINIMA), Academic Medical Center of the University of Amsterdam, Amsterdam, The Netherlands; 4 Present address: Crucell Holland BV, Leiden, The Netherlands

**Keywords:** HIV-1, Viremic long-term non-progressors, Immune activation, Nef function

## Abstract

**Background:**

A small minority of HIV-1-infected individuals show low levels of immune activation and do not develop immunodeficiency despite high viral loads. Since the accessory viral Nef protein modulates T cell activation and plays a key role in the pathogenesis of AIDS, we investigated whether specific properties of Nef may be associated with this highly unusual clinical outcome of HIV-1 infection.

**Findings:**

Comprehensive functional analyses of sequential HIV-1 strains from three viremic long-term non-progressors (VNP) showed that they encode full-length Nef proteins that are capable of modulating CD4, CD28, CD8ß, MHC-I and CD74 cell surface expression. Similar to Nef proteins from HIV-1-infected individuals with progressive infection (P-Nefs) and unlike Nefs from simian immunodeficiency viruses (SIVs) that do not cause chronic immune activation and disease in their natural simian hosts, VNP-Nefs were generally unable to down-modulate TCR-CD3 cell surface expression to block T cell activation and apoptosis. On average, VNP-Nefs suppressed NF-AT activation less effectively than P-Nefs and were slightly less active in enhancing NF-κB activity. Finally, we found that VNP-Nefs increased virion infectivity and enhanced HIV-1 replication and cytopathicity in primary human cells and in *ex vivo* infected lymphoid tissues.

**Conclusions:**

Our results show that *nef* alleles from VNPs and progressors of HIV-1 infection show only modest differences in established functions. Thus, the lack of chronic immune activation and disease progression in HIV-1-infected VNPs is apparently not associated with unusual functional properties of the accessory viral Nef protein.

## Findings

High viral loads are almost invariably associated with chronic inflammation and progression to AIDS in HIV-1-infected individuals. In contrast, some non-human primates (NHPs) that are naturally infected with SIVs, such as sooty mangabeys (SMs) or African green monkeys (AGMs), show low levels of immune activation and do not develop disease despite high levels of viral replication [1,2]. Recent data show that a small minority (<1%) of highly viremic HIV-1-infected individuals, so called viremic long-term non-progressors (VNP), show a similar phenotype and remain asymptomatic with low levels of inflammation and high CD4^+^ T cell counts [3].

It is poorly understood why VNPs can tolerate high levels of HIV-1 replication. It has been reported, however, that they show lower levels of proliferating and activated T cells than progressing individuals with similar viral loads [3]. Here, we examined whether specific functional properties of the accessory viral Nef protein may contribute to the low levels of T cell activation and the lack of disease progression in VNPs. Nef is a multi-functional manipulator of the viral host cell that facilitates viral immune evasion and is critical for efficient viral replication and disease progression in HIV-1-infected individuals [4,5]. Primate lentiviral Nef proteins generally down-modulate CD4 and MHC-I from the cell surface and enhance viral infectivity and replication [4,5]. They differ fundamentally, however, in their effect on the responsiveness of virally infected T cells to stimulation. HIV-1 Nef proteins may render infected T cells hyper-responsive to stimulation and promote the induction of cellular transcription factors, activation markers and inflammatory cytokines [6-9]. In contrast, most primate lentiviral Nefs block T cell activation by down-modulation of TCR-CD3 and CD28 from the cell surface [10]. These latter Nef functions are highly conserved in sooty mangabeys that are naturally infected with SIVsmm [11] and may play a protective role *in vivo* since efficient modulation of TCR-CD3 and CD28 correlates with high and stable CD4^+^ T cell counts in natural SIVsmm infection [12] and in viremic HIV-2-infected individuals [13].

**Figure 1:**
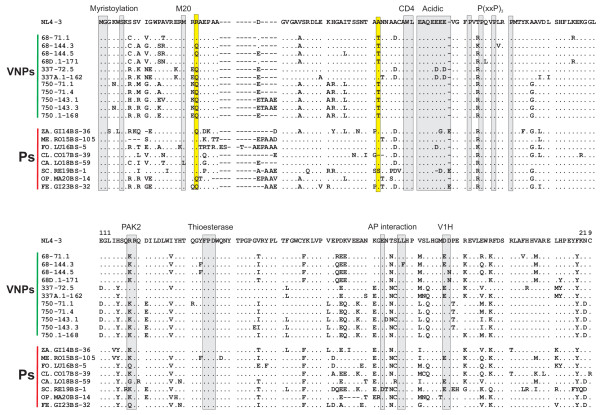
**Amino acid alignment of VNP- and P-Nef proteins.** Nef alleles from VNPs and Ps are compared. Some conserved sequence elements in Nef, including the N-terminal myristoylation signal, the M20 and WL residues (involved in MHC-I or CD4 down-modulation, respectively), the acidic, proline-rich and diarginine motifs, a thioesterase binding site, a C-proximal adaptor-protein (AP) interaction site and a diacidic putative V1H binding site are indicated. Residues 22 and 62 are highlighted yellow. Dots specify identity with the NL4-3 Nef sequence; dashes indicate gaps introduced to optimize the alignment.

To examine their functional properties, we PCR-amplified *nef* genes from three previously described HIV-1-infected VNPs from the Amsterdam Cohort Studies on HIV infection and AIDS [3] obtained after six to 14 years of documented HIV-1 infection. For comparison, we utilized *nef* alleles amplified from plasma samples obtained from eight individuals with progressing HIV-1 infection (P) and seven previously characterized HIV-1, HIV-2, SIVmac and SIVsmm *nef* alleles (Additional file 1: Table S1). It has previously been shown that SIV Nefs modulate various receptors and enhance viral infectivity and replication in a species-independent manner [10,14]. Between four and ten *nef* alleles were sequenced per VNP and P sample and those predicting amino acid sequences that were identical or almost identical to the respective patient and time point-specific consensus sequence and thus most representative were selected for further analysis (Figure 1). All *nef* alleles encoded and expressed full-length proteins (Additional file 1: Figures S1) and previously defined functional domains were conserved (Figure 1), suggesting that Nef proteins from both groups of HIV-1-infected individuals are functionally active. No specific sequence signatures were observed in VNP-Nefs, although they frequently contained 22Q (9/11) and 62T (9/11), whereas those from progressing individuals contained 22R (5/8) and 62A (6/8; numbers indicate positions in the Nef alignment shown in Figure 1). Given the different cohorts from which the VNP and P samples originated, these are likely to be differences in founder sequences.

**Figure 2:**
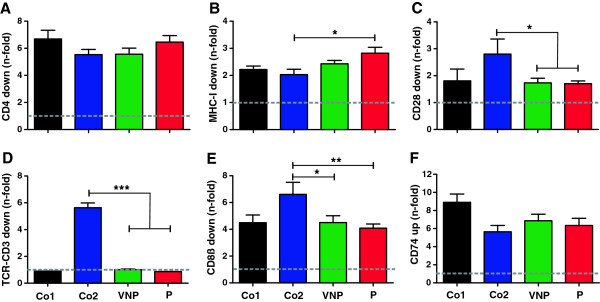
**No group-specific difference of Nef-mediated regulation of surface receptors. (A-F)** Quantitative assessment of flow cytometric analysis of Nef-mediated modulation of **(A)** CD4 and **(B)** MHC-I in primary CD4^+^ T cells, **(C)** CD28 and **(D)** TCR-CD3 in PBMCs, **(E)** CD8β in CEM A2-CD8β fusion cells and **(F)** CD74 (Ii) in THP-1 cells transduced with HIV-1 recombinants expressing eGFP alone (*nef-*) or together with control or patient-derived *nef* alleles. A *vpu* and *env* defective HIV-1 backbone was used to measure the effect of Nef on CD4 surface expression. Given are *n-fold* modulation of cell surface expression compared to the *nef*-defective control HIV-1 construct (average values ± standard errors) derived from three independent experiments. *Nef* alleles were derived from the HIV-1 NL4-3, NA7 and JR-CSF molecular clones (Co1, black); SIVmac239, HIV-2 BEN, HIV-2 60415 K, SIVsmm FWr1 and FFm1 Nefs (Co2, blue), VNPs (n = 11, green) and Ps (n = 8, red). Co, control; *, p < 0.05; **, p < 0.01; ***, p < 0.001.

For functional analyses all *nef* alleles shown in Figure 1 were cloned into an HIV-1 NL4-3 proviral construct that co-expresses Nef and eGFP via an internal ribosomal entry site [14]. Viral particles were generated by co-transfection of 293T cells with the HIV-1 *nef*-IRES-eGFP proviral constructs and a plasmid (pHIT-G) expressing the vesicular stomatitis virus G glycoprotein and used for transduction of peripheral blood mononuclear cells (PBMCs) or CD4^+^ T cells as previously described [10,12]. Flow cytometric analyses performed at two days post-transduction showed that *nef* alleles from both VNPs and Ps down-modulated CD4 and (to a lesser extent) MHC-I and CD28 from the cell surface (Figures 2A-C and Additional file 1: Figure S2). VNP and P Nefs did not show significant differences in receptor modulation but were significantly less active in down-modulating CD28 than HIV-2 and SIV Nefs (Co2) (Figure 2C). Moreover, none of the patient-derived and control HIV-1 Nefs was capable of down-modulating TCR-CD3, whereas HIV-2 and SIV Nefs were highly effective (Figure 2D).

In addition to reducing the lysis of HIV-1-infected cells by cytotoxic T lymphocytes (CTL) by removing MHC-I from the cell surface, Nef is also able to impair CTL function directly by down-modulation of CD8β [15,16]. Analyses performed in CEM cells stably expressing A2-CD8β fusions [15] confirmed that CD8β down-modulation is conserved between *nef* alleles derived from both groups of HIV-1-infected individuals (Figures 2E and Additional file 1: FigureS2). Unexpectedly, HIV-2, SIVsmm and SIVmac Nefs that all belong to the same lineage of primate lentiviruses were significantly more active than the HIV-1 control Nefs (Figure 2E). Nef may also impair MHC-II-dependent antigen-presentation by up-regulating surface expression of the invariant chain (CD74) [17,18]. Potentially, inefficient activation of T cells by antigen-presenting cells (APCs) due to potent up-modulation of CD74 may reduce the levels of immune activation. We found, however, that VNP- and P-Nefs did not differ significantly in their ability to enhance CD74 cell surface expression in infected THP-1 cells (Figure 2F). Up-modulation of CD74 was reduced for *nef* alleles obtained later during infection of VNPs 68 and 337, whereas the ability of VNP-Nefs to modulate other receptors did not change significantly throughout the course of infection (Additional file 1: Figure S3). Whether or not the ability of Nef to suppress MHC-II antigen presentation by up-regulation of CD74 may be modulated throughout the course of HIV-1 infection and if this Nef function plays a role in the pathogenesis of AIDS remains to be analyzed in larger patient cohorts.

**Figure 3:**
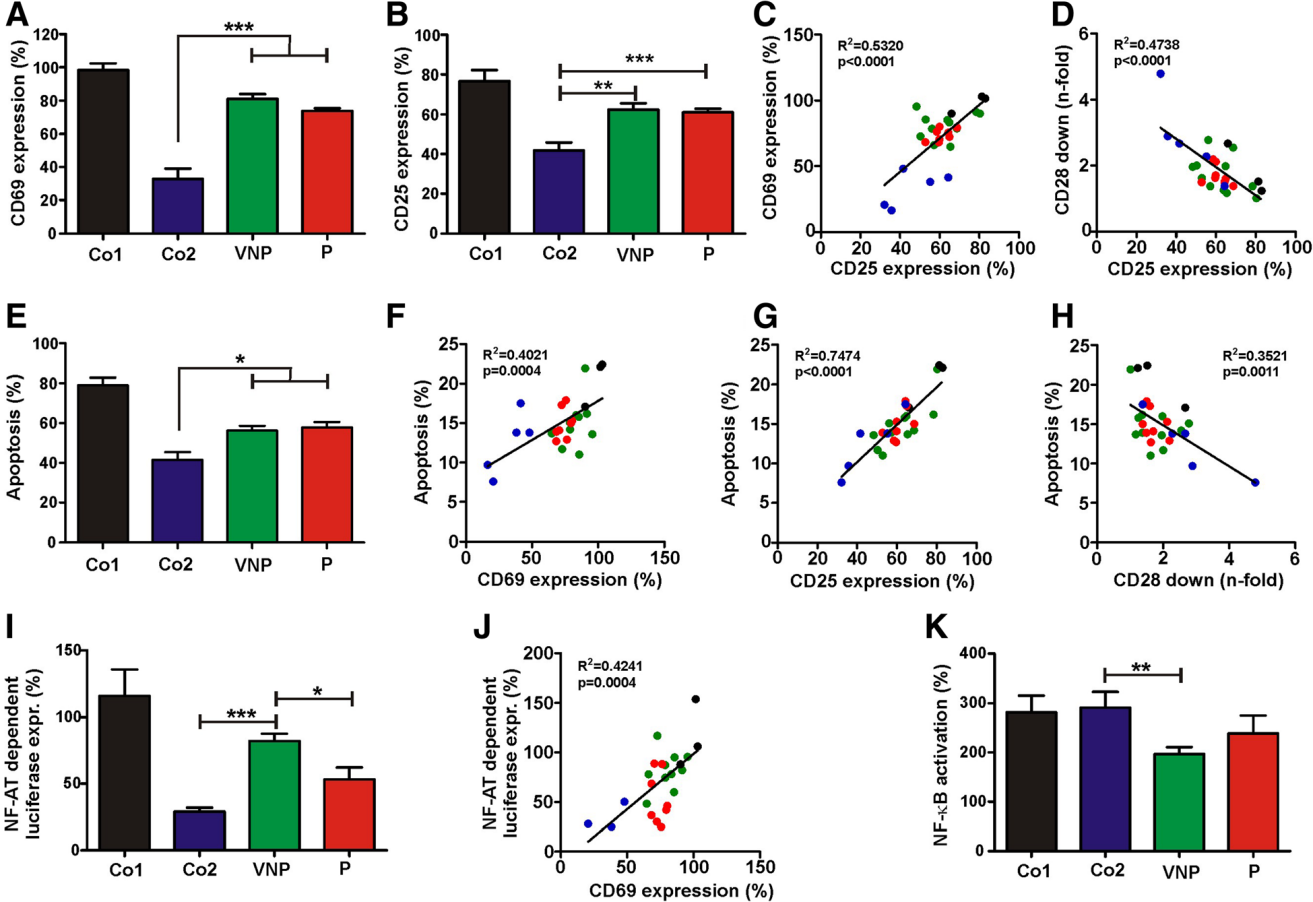
**VNP-Nefs cannot prevent activation-induced apoptosis of infected cells. (A, B)** Expression of **(A)** CD69 and **(B)** CD25 at one or two days, respectively, after the second PHA stimulation in PBMCs transduced with HIV-1 IRES/eGFP constructs expressing the indicated groups of Nefs. **(C, D)** Correlations between expression of CD25 and **(C)** CD69 or **(D)** Nef-mediated CD28 down-modulation. **(E)** Quantitative assessment of levels of apoptosis two days post-stimulation in HIV-1-infected PBMCs. **(F-H)** Correlation between apoptosis and expression of **(F)** CD69 and **(G)** CD25 or **(H)** CD28 down-modulation by the respective Nefs. **(I)** PHA-induced NF-AT-dependent luciferase activity in HIV-1 infected Jurkat cells stably transfected with an NF-AT-dependent reporter gene [9]. **(J)** Correlation between CD69 expression and NF-AT-dependent luciferase expression. **(K)** Nef-mediated activation of NF-κB in 293Ts co-transfected with a NF-κB-dependent firefly luciferase construct, a pTAL promoter gaussia luciferase construct and an expression vector expressing the various Nef proteins in the presence of TNFα. The assay was performed as described elsewhere [21]. Panels A, B, E, I and K give average values (±SD) relative to those obtained with the *nef*-defective control HIV-1 construct and were derived from three independent experiments. Refer to the legend to Figure 2 for abbreviations, symbols, color coding and *nef* alleles analyzed.

The above mentioned results demonstrated that the low levels of immune activation in VNPs were not associated with an increased activity of Nef to modulate receptors involved in the activation of T cells by APCs. It is known, however, that the HIV-1 Nef may also affect T cell activation by modulating downstream signaling pathways [19,20]. To directly examine the effects of Nef on the responsiveness of primary human cells to activation, we infected PBMCs with various HIV-1 IRES/eGFP constructs and stimulated them by PHA treatment as described previously [10]. The results showed that virally infected cells expressing VNP- and P-Nefs expressed higher surface levels of CD69 and CD25 than cells producing HIV-2 or SIV Nefs (Figure 3A, B). Expression of the early and late T cell activation markers correlated with one another (Figure 3C) and inversely with the efficiency of Nef-mediated modulation of CD28 (Figure 3D). Similarly, the levels of apoptosis were higher in HIV-1-infected PBMCs expressing VNP- and P-Nefs, compared to those expressing SIV or HIV-2 Nefs (Figure 3E) and correlated directly with the expression of T cell activation markers and inversely (albeit imperfectly) with CD28 cell surface expression (Figure 3F-H). These results are in agreement with previous studies showing that Nef-mediated down-modulation of TCR-CD3 and (to a lesser extent) CD28 suppresses the responsiveness of virally infected T cells to stimulation and activation-induced cell death [10,12,13].

It has been reported that HIV-1 Nef may increase activation of the nuclear factors of activated T cells and kappa B (NF-AT and NF-κB), respectively [9]. Both of these transcription factors play key roles in innate immunity and inflammatory responses [22,23] and may thus affect the levels of infection-associated immune activation. Measurements of the impact of Nef on NF-AT-dependent luciferase expression in stably transfected Jurkat T cells [9] showed that HIV-1 Nefs were generally associated with higher levels of NF-AT activation than HIV-2 and SIV Nefs (Figure 3I, J). While this was expected from published data [9], it came as a surprise that P-Nefs suppressed NF-AT activation more efficiently than VNP-Nefs. In contrast, P-Nefs trended towards association with higher levels of NF-κB activation than VNP-Nefs, although this difference failed to reach significance (Figure 3K). Notably, we observed that *nef* alleles from the three T cell line adapted molecular clones of HIV-1 were associated with higher levels of CD69 (p = 0.0013) and CD25 (p = 0.0012) expression, apoptosis (p = 0.0002) and NF-AT activation (p = 0.0111) than primary patient-derived *nef* genes (Figure 3). It is also noteworthy that HIV-2 and SIV Nefs efficiently suppressed induction of NF-AT but were particularly active in stimulating NF-κB (Figure 3I, K). Thus, these primate lentiviruses may efficiently activate the viral LTR via its NF-κB containing core enhancer element but avoid NF-AT dependent induction of immune response genes.

**Figure 4:**
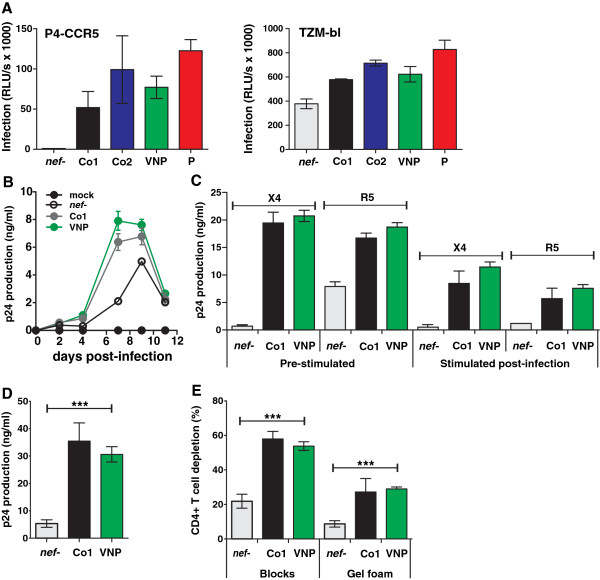
**VNP Nefs enhance virion infectivity and viral replication. (A)** P4-CCR5 (left) and TZM-bl (right) cells were infected with recombinant HIV-1 IRES-EGFP constructs expressing the indicated groups of *nef* alleles. Infections were performed in triplicate with two independent virus stocks. All panels give average values ± SD. RLU, relative light units. **(B)** Average levels of p24 antigen and **(C)** cumulative p24 production by PBMCs over 11 days of culture. PBMCs were either stimulated with PHA (1 μg/ml) for 3 days prior to infection with the X4-tropic HIV-1 NL4-3 clone or an R5-tropic derivative thereof [28] or infected immediately after isolation and stimulated three days later. Supernatants were collected at 2- or 3-day intervals, and productive HIV-1 infection was assessed by measuring p24 antigen content. The values were derived from three independent experiments. **(D)** Cumulative virus production in human lymphoid tissue (HLT) infected ex vivo. **(E)** Depletion of CD4+ T cells in HLT infected ex vivo. Tissues from six donors were infected with X4 HIV-1 NL4-3 expressing the indicated *nef* alleles, and cumulative p24 production by the tissue blocks over 15 days or CD4+ T-cell depletion at the end of culture was determined as described previously [25,27]. Refer to the legend to Figure 2 for abbreviations, symbols, color coding and *nef* alleles analyzed.

Finally, we examined the ability of VNP-Nefs to enhance HIV-1 infection, replication and cytopathicity. Infection of P4-CCR5 and TZM-bl indicator cells [24] with virus stocks containing normalized quantities of p24 antigen derived from 293T cells transiently transfected with the different proviral constructs [24], showed that most VNP- and P-Nefs enhanced virion infectivity, albeit with variable efficacy (Figure 4A, Additional file 1: Figure S3). In agreement with published data [25] the magnitude of the Nef effect on HIV-1 infection was much higher in P4-CCR5 cells than in TZM-bl cells that are highly susceptible to infection. To determine the effect of Nef on HIV-1 replication in primary human cells, PBMCs were infected with HIV-1 constructs and virus production in the supernatant was determined as described previously [26]. In agreement with the high viral loads, VNP-Nefs increased virus production (Figure 4B), irrespectively of the viral coreceptor tropism, and of whether or not the PBMCs were pre-stimulated or stimulated 3 days after infection (Figure 4C). Since PBMC cultures may not faithfully recapitulate the viral phenotype *in vivo*, we also determined the levels of viral replication and CD4^+^ T cell depletion in *ex vivo* human tonsillary tissues that allow determination of the cytopathicity and replication capacity of HIV-1 without exogenous stimulation [27]. Since the number of cultures that could be set up per tonsil was limited, we only compared the effect of the VNP-Nefs with the HIV-1 control Nefs. We found that HIV-1 constructs expressing VNP-Nefs replicated efficiently in human lymphoid tissue (HLT) and depleted CD4^+^ T cells as effectively as otherwise isogenic viral strains expressing the control NL4-3 and NA7 Nefs (Figure 4D, E). These results are in agreement with the previous finding that biologically cloned HIV-1 isolates from these three VNPs are cytopathic and replication competent in *ex vivo* infected human lymphoid tissue [3].

In conclusion, low levels of chronic inflammation and lack of disease progression in VNPs are not due to efficient Nef-mediated suppression of T cell activation. The possible relevance and significance of the modest differences in modulation of NF-AT and NF-κB by VNP- and P-Nefs observed in the present study needs to be further examined in larger patient cohorts. Notably, our data suggest that *nef* alleles derived from primary HIV-1 strains but not those of T cell line adapted molecular clones of HIV-1 may suppress T cell activation, albeit much less efficiently than HIV-2 and SIV *nef* genes that down-modulate TCR-CD3. Thus, our results further underline the necessity to use primary *nef* alleles to avoid *in vitro* artifacts. Finally, our findings suggest that host factors rather than specific viral properties may allow VNPs to avoid harmful chronic immune activation by HIV-1 replication.

## Competing interests

The authors declare no conflict of interest.

## Authors’ contributions

AH performed most experiments. DC and AvW contributed reagents. AH and FK designed the study, performed the statistical analysis, and wrote the manuscript. All authors read and approved the final manuscript.

## Supplementary Material

Additional file 1 **Overview on *nef *alleles analyzed. Figure S1.** Expression of Nef proteins. Western blot analysis of lysates from 293T cells transfected with pCGCG vectors expressing AU-1-tagged versions of the indicated Nef proteins. Lysates were probed with an anti AU-1 monoclonal antibody (Covance), anti β-actin polyclonal antibody (Abcam) and anti GFP polyclonal antibody (Abcam). **Figure S2.** Modulation of various receptors by VNP- and P-Nefs. Primary CD4+ T cells were transduced with NL4-3 constructs coexpressing the indicated *nef* alleles and GFP and assayed by FACS. CEM cells expressing A2-CD8β fusions and THP-1 cells were utilized to examine modulation of CD8ß and CD74 expression, respectively. The ranges of eGFP expression used to calculate receptor modulation in Figure 1 are indicated. **Figure S3.** Activity of VNP-Nefs throughout the course of infection. (A-F) Quantitative assessment of Nef-mediated modulation of (A) CD4, (B) MHC-I, (C) CD28 and (D) TCR-CD3 in primary cells, (E) CD8β in CEM A2-CD8β fusion cells and (F) CD74 (Ii) in THP-1 cells transduced with HIV-1 recombinants expressing eGFP alone (*nef*-) or together with various *nef* alleles. (G to I) Quantitative analysis of (G) CD69 expression, (H) CD25 expression or (I) apoptosis levels in transduced PBMCs. (J) PHA-induced NF-AT-dependent luciferase activity obtained from transduced Jurkat cells stably transfected with an NF-AT-dependent reporter gene. (K) Analysis of Nef-mediated activation of NF-κB in 293Ts co-transfected with a NF-κB-dependent firefly luciferase construct, a pTAL promoter gaussia luciferase construct (to normalize) and Nef expression vectors in the presence of TNFα. (L) Nef-mediated enhancement of infectivity in P4-CCR5 cells. Given are average values ±SEM derived from multiple experiments of Nefs from HIV-1 NA7 (white), HIV-2 BEN (blue), VNP 68 (light green), VNP 337 (middle green) and VNP 750 (dark green). Numbers below bars provide month of sampling after the estimated data of primary infection. Click here for file
