# Supplementary material for: Viremic long-term nonprogressive HIV-1 infection is not associated with abnormalities in known Nef functions
Source: Retrovirology. 2014 Feb 4;11:13. doi: 10.1186/1742-4690-11-13 (PMC3927655; doi:10.1186/1742-4690-11-13)
Supplement: Additional file 1 — Overview on nef alleles analyzed. Figure S1. Expression of Nef proteins. Western blot analysis of lysates from 293T cells transfected with pCGCG vectors expressing AU-1-tagged versions of the indicated Nef proteins. Lysates were probed with an anti AU-1 monoclonal antibody (Covance), anti β-actin polyclonal antibody (Abcam) and anti GFP polyclonal antibody (Abcam). Figure S2. Modulation of various receptors by VNP- and P-Nefs. Primary CD4+ T cells were transduced with NL4-3 constructs coexpressing the indicated nef alleles and GFP and assayed by FACS. CEM cells expressing A2-CD8β fusions and THP-1 cells were utilized to examine modulation of CD8ß and CD74 expression, respectively. The ranges of eGFP expression used to calculate receptor modulation in Figure 1 are indicated. Figure S3. Activity of VNP-Nefs throughout the course of infection. (A-F) Quantitative assessment of Nef-mediated modulation of (A) CD4, (B) MHC-I, (C) CD28 and (D) TCR-CD3 in primary cells, (E) CD8β in CEM A2-CD8β fusion cells and (F) CD74 (Ii) in THP-1 cells transduced with HIV-1 recombinants expressing eGFP alone (nef-) or together with various nef alleles. (G to I) Quantitative analysis of (G) CD69 expression, (H) CD25 expression or (I) apoptosis levels in transduced PBMCs. (J) PHA-induced NF-AT-dependent luciferase activity obtained from transduced Jurkat cells stably transfected with an NF-AT-dependent reporter gene. (K) Analysis of Nef-mediated activation of NF-κB in 293Ts co-transfected with a NF-κB-dependent firefly luciferase construct, a pTAL promoter gaussia luciferase construct (to normalize) and Nef expression vectors in the presence of TNFα. (L) Nef-mediated enhancement of infectivity in P4-CCR5 cells. Given are average values ±SEM derived from multiple experiments of Nefs from HIV-1 NA7 (white), HIV-2 BEN (blue), VNP 68 (light green), VNP 337 (middle green) and VNP 750 (dark green). Numbers below bars provide month of sampling after the estimated data of primary infection. [file 1742-4690-11-13-S1.pdf]

Supplementary data

Viremic Long-Term Nonprogressive HIV-1 Infection is not associated with Abnormalities in Nef Function

Anke Heigele, David Camerini, Angelique van 't Wout, and Frank Kirchhoff[1*]

The supplementary data contain one supplementary Table, three supplementary Figure and supplementary references.

Table S1. Overview on *nef* alleles analyzed.

Clone	Group	Cohort	infection time (months)	Source	Accession*	References
NL4-3	Control	n.a.	n.a.	human T cell line	M19921	[29]
NA7	Control	n.a.	n.a.	human PBMC	DQ242535	[30]
JR-CSF	Control	n.a.	n.a.	human PBMC	M38429	[31]
750-71.1	VNP	Amsterdam	71	human T cells	n.a.	[3]
750-71.4	VNP	Amsterdam	71	human T cells	n.a.	[3]
750-143.1	VNP	Amsterdam	143	human T cells	n.a.	[3]
750-143.3	VNP	Amsterdam	143	human T cells	n.a.	[3]
750.1-168	VNP	Amsterdam	168	human plasma	n.a.	[3]
337-72.5	VNP	Amsterdam	72	human T cells	n.a.	[3]
337A.1-162	VNP	Amsterdam	162	human plasma	n.a.	[3]
68-71.1	VNP	Amsterdam	71	human T cells	n.a.	[3]
68-144.3	VNP	Amsterdam	144	human T cells	n.a.	[3]
68-144.5	VNP	Amsterdam	144	human T cells	n.a.	[3]
68D.1-171	VNP	Amsterdam	171	human plasma	n.a.	[3]
ZA.GI14BS-36	P	Brescia	36	human plasma	n.a.	(unpublished)
ME.RO15BS-105	P	Brescia	105	human plasma	n.a.	(unpublished)
FO.LU16BS-5	P	Brescia	5	human plasma	n.a.	(unpublished)
CL.CO17BS-39	P	Brescia	39	human plasma	n.a.	(unpublished)
CA.LO18BS-59	P	Brescia	59	human plasma	n.a.	(unpublished)
SC.RE19BS-1	P	Brescia	1	human plasma	n.a.	(unpublished)
OP.MA20BS-14	P	Brescia	14	human plasma	n.a.	(unpublished)
FE.GI23BS-32	P	Brescia	32	human plasma	n.a.	(unpublished)
HIV-2 BEN	Control	n.a.	n.a.	human T cells	M30502	[32]
HIV-2 60415K	Control	n.a.	n.a.	human PBMC	DQ092764	[33]
SIVmac239	Control	n.a.	n.a.	RM PBMCs	M33262	[34]
SIVsmm FWr1	Control	n.a.	n.a.	SM blood	DQ092758	[33]
SIVsmm FFm1	Control	n.a.	n.a.	SM blood	DQ092762	[33]

* Sequences will be submitted to the GenBank sequence database and accession numbers will be provided upon acceptance of the manuscript. N.a., not applicable or not available.

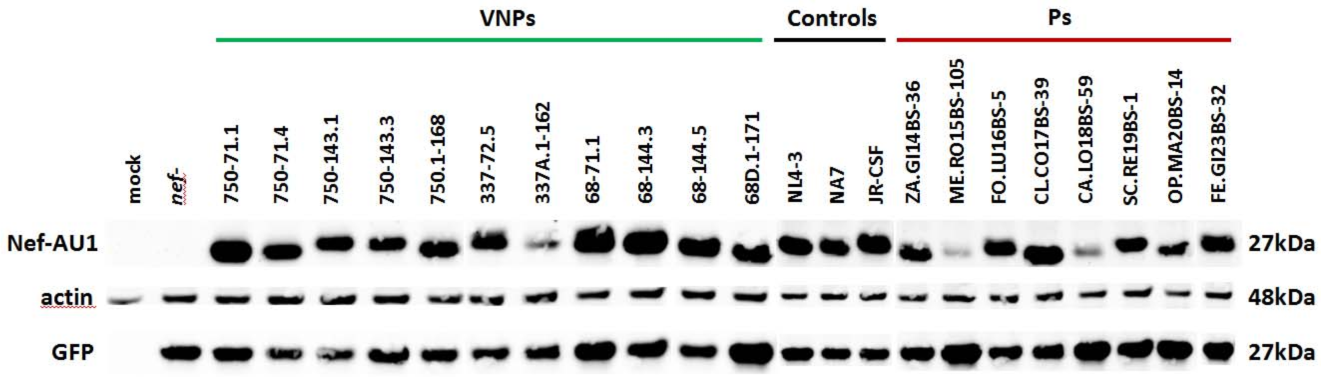

Figure S1. Expression of VNP- and P-Nef proteins. Western blot analysis of cell lysates following transfection of 293T cells with pCGCG vectors expressing AU-1-tagged versions of the indicated Nef proteins. Lysates were probed with an anti AU-1 monoclonal antibody (Covance), anti β-actin polyclonal antibody (Abcam) and anti GFP polyclonal antibody (Abcam) to determine protein expression levels.

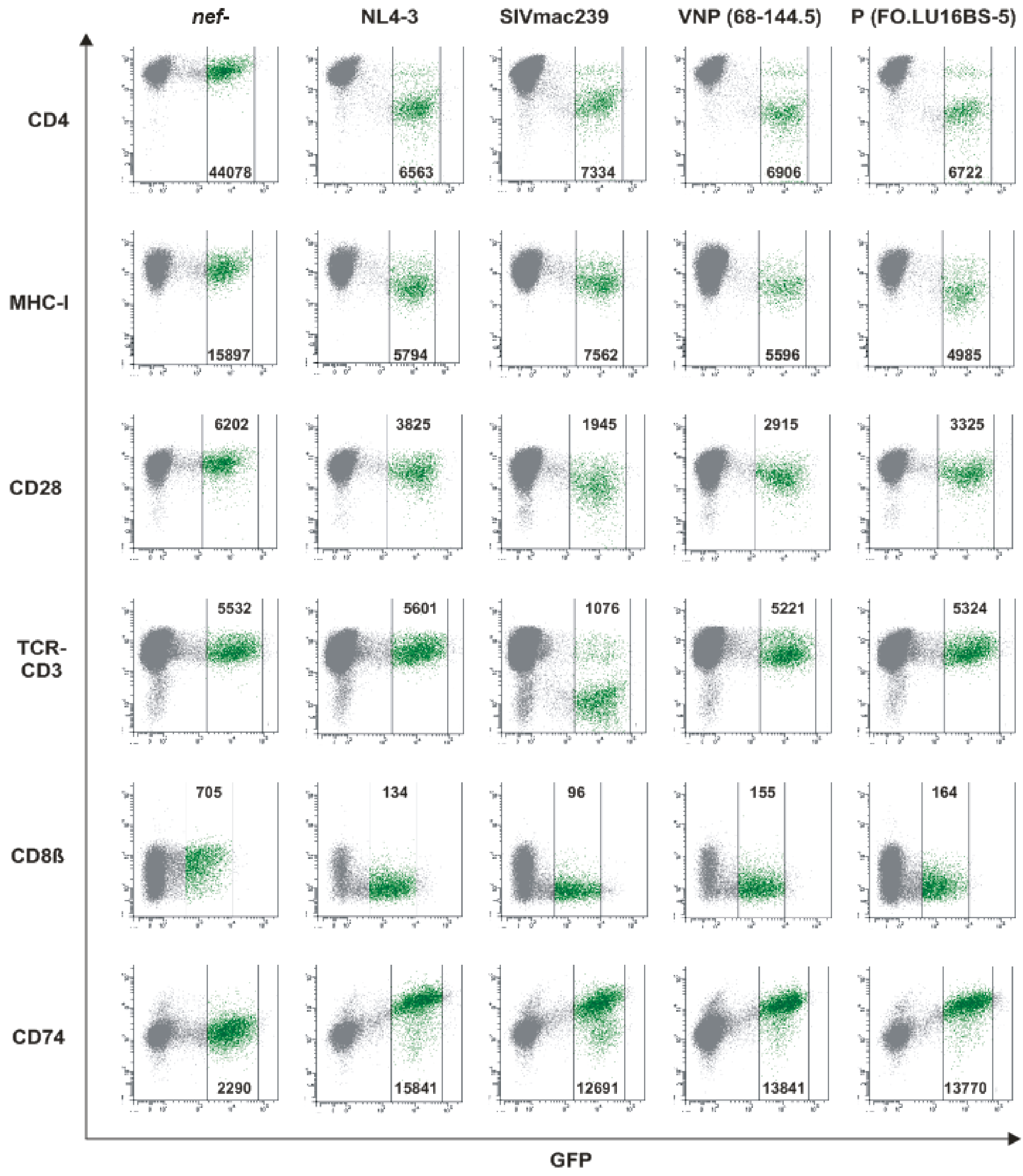

Figure S2. Modulation of various receptors by VNP- and P-Nef proteins. To monitor effects of CD4, MHC-I, CD28 and TCR-CD3 primary CD4+ T cells were transduced with HIV-1 NL4-3 constructs coexpressing the indicated *nef* alleles and GFP and assayed by flow cytometric analyses. CEM cells expressing A2-CD8β fusions and THP-1 cells were utilized to examine Nef-mediated modulation of CD8ß and CD74 expression, respectively. The ranges of eGFP expression used to calculate receptor modulation in Figure 1 are indicated.

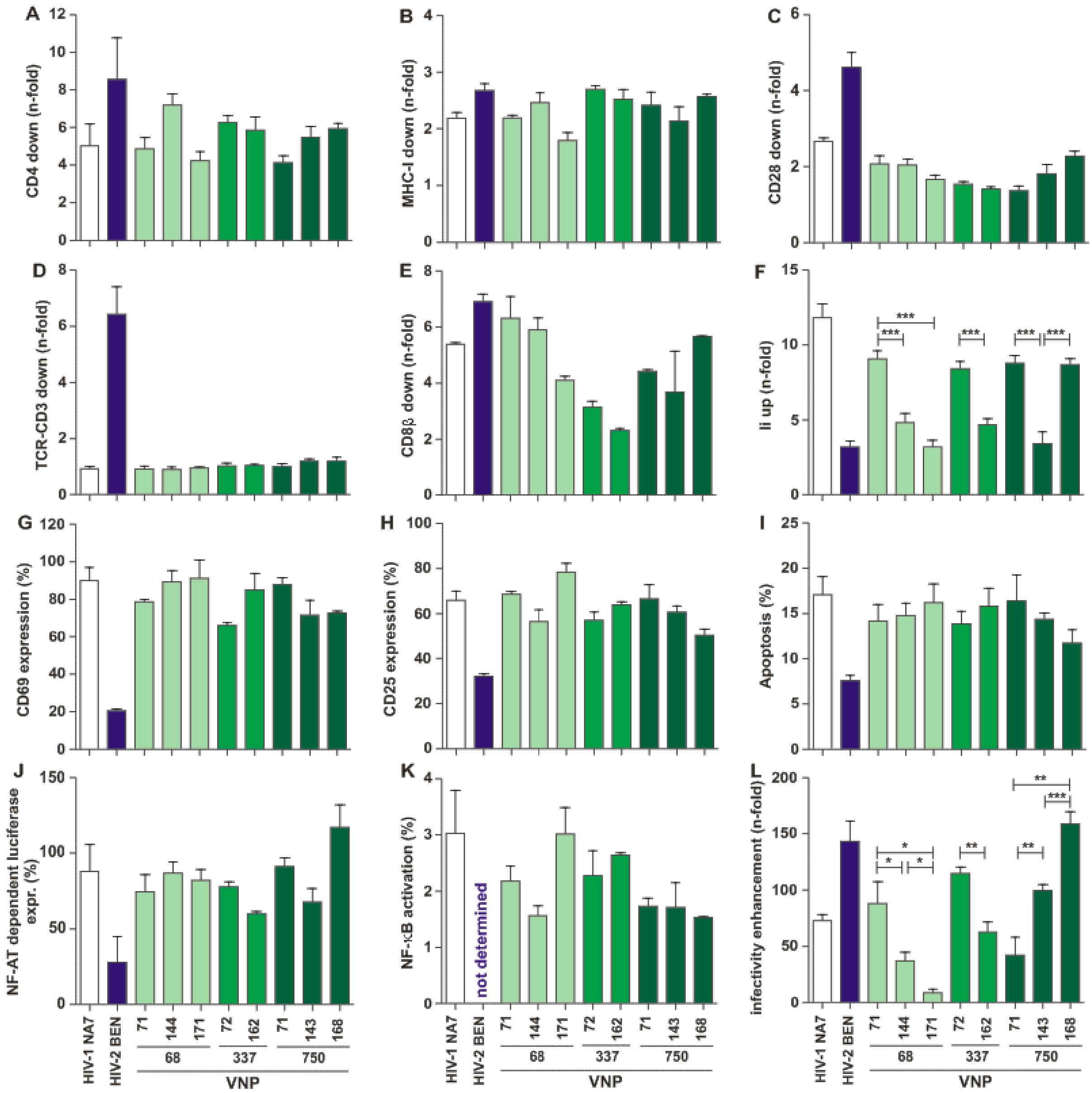

Figure S3. Activity of VNP-Nefs throughout the course of infection. (A-F) Quantitative assessment of Nef-mediated modulation of (A) CD4, (B) MHC-I, (C) CD28 and (D) TCR-CD3 in primary cells, (E) CD8β in CEM A2-CD8β fusion cells and (F) CD74 (Ii) in THP-1 cells transduced with HIV-1 recombinants expressing eGFP alone (*nef*-) or together with control or VNP-derived *nef* alleles. (G to I) Quantitative analysis of (G) CD69 expression, (H) CD25 expression or (I) apoptosis levels in transduced PBMCs. (J) PHA-induced NF-AT-dependent luciferase activity obtained from transduced Jurkat cells stably transfected with an NF-AT-dependent reporter gene. (K) Analysis of Nef-mediated activation of NF-κB in 293Ts co-transfected with a NF-κB-dependent firefly luciferase construct, a pTAL promoter gaussia luciferase construct (to normalize) and an expression vector expressing the various Nef proteins in the presence of TNFα. (L) Nef-mediated enhancement of infectivity in P4-CCR5 indicator cells. Given are average values ± standard errors derived from multiple experiments of Nefs from HIV-1 T cell line adapted molecular clone NA7 (white), HIV-2 BEN (blue), VNP 68 (light green), VNP 337 (middle green) and VNP 750 (dark green). Numbers below bars provide month of sampling after the estimated data of primary infection.

Supplementary references:

29. Adachi A, Gendelman HE, Koenig S, Folks T, Willey R, Rabson A, Martin MA: **Production of acquired immunodeficiency syndrome-associated retrovirus in human and nonhuman cells transfected with an infectious molecular clone.** *J Virol* 1986, **59:**284-291.

30. Mariani R, Skowronski J: **CD4 down-regulation by nef alleles isolated from human immunodeficiency virus type 1-infected individuals.** *Proc Natl Acad Sci USA* 1993, **90:**5549-5553.

31. O´Brien WA, Koyanaqu Y, Namazie A, Zhao JQ, Diaqne A, Idler K, Zack JA, Chen IS: **HIV-1 tropism for mononuclear phagocytes can be determined by regions of gp120 outside the CD4-binding domain.** *Nature* 1990, **348:**69-73.

32. Kirchhoff F, Jentsch KD, Bachmann B, Stuke A, Laloux C, Lüke W, Stahl-Hennig C, Schneider J, Nieselt K, Eigen M, Hunsmann G: **A novel proviral clone of HIV-2: Biological and phylogenetic relationship to other primate immunodeficiency viruses.** *Virology* 1990, **177:**305-311.

33. Münch J, Schindler M, Wildum S, Rücker E, Bailer N, Knoop V, Novembre FJ, Kirchhoff F: **Primary sooty mangabey simian immunodeficiency virus and human immunodeficiency virus type 2 nef alleles modulate cell surface expression of various human receptors and enhance viral infectivity and replication.** *J Virol* 2005, **79:**10547-10560.

34. Kestler H, Kodama T, Ringler D, Marthas M, Pedersen N, Lackner A, Regier D, Sehgal P, Daniel M, King N, Desrosiers R: **Induction of AIDS in rhesus monkeys by molecularly cloned simian immunodeficiency virus.** *Science* 1990, **248:**1109-1112.
